# Molecular mechanism of valine and its metabolite in improving triglyceride synthesis of porcine intestinal epithelial cells

**DOI:** 10.1038/s41598-023-30036-w

**Published:** 2023-02-20

**Authors:** Mengmeng Xu, Long Che, Lizhu Niu, Liuzhen Wang, Mengyun Li, Dongfeng Jiang, Hongyu Deng, Wen Chen, Zongyong Jiang

**Affiliations:** 1 College of Animal Science and Technology, Henan University of Animal Husbandry and Economy, No.6 North Longzihu Road, Zhengdong New District, Zhengzhou, 450046 Henan China; 2 College of Livestock Husbandry and Veterinary Engineering, Henan Agricultural University, No. 15 Longzi Lake University Campus, Zhengzhou, 450046 China; 3 Institute of Animal Science, Guangdong Academy of Agricultural Sciences, Guangzhou, 510640 Guangdong China

**Keywords:** Fat metabolism, Animal physiology

## Abstract

An insufficient energy supply to intestinal epithelial cells decreases production performance in weaned piglets. Triglycerides are the main energy source for intestinal epithelial cells in piglets. The present study aimed to investigate the effects and mechanisms of valine supplementation on triglyceride synthesis in porcine intestinal epithelial **(**IPEC-J2) cells. Valine supplementation in the medium significantly increased the content of triglycerides, fat droplets, and long-chain fatty acids (C17:0, C18:0, C20:0, C18:1, C20:1, and C22:1) (*P* < 0.05). Valine metabolite (3-hydroxyisobutyrate [3-HIB]) concentration increased significantly in the valine-supplemented group (*P* < 0.05). Silencing of the 3-HIB synthase enzyme 3-hydroxyisobutyryl-CoA hydrolase (HIBCH) in IPEC-J2 cells significantly reduced the triglyceride concentration and lipid droplet synthesis. Further studies found that 3-HIB supplementation in the medium significantly increased the concentration of triglycerides, lipid droplets, and unsaturated fatty acids (C16:1, C18:1, C18:2, C18:3, C20:3, C20:4, and C20:5) (*P* < 0.05) by upregulating the expression of proteins involved in fatty acid transport (CD36) and fatty acid binding protein 3 (FABP3) or triglyceride synthesis (DGAT1) (*P* < 0.05), indicating that 3-HIB mediates valine-enhanced triglyceride synthesis in IPEC-J2 cells. In conclusion, our results demonstrated that valine enhanced triglyceride synthesis in IPEC-J2 cells via increasing the 3-HIB concentration, which may promote fatty acid transport via upregulation of proteins related to fatty acid transporter. These findings provide new insights into the mechanisms through which valine participates in lipid metabolism.

## Introduction

Weaning stress in piglets changes intestinal morphology and decreases digestive capacity and absorption, leading to insufficient energy supply by intestinal cells^1–3^. Changes in intestinal structure and function reduce growth and increase the diarrhea rate in piglets^4^. Dietary lipids provide energy and essential fatty acids to humans and animals. Adding oil to piglet diet can improve piglet health and production performance^5^; however, weaned piglets cannot meet the energy demand for rapid growth owing to low feed intake^6^. Meanwhile, the supplementation of oil in the diet easily leads to lipid peroxidation and oxidative stress in piglets^7^, and feeding costs will also increase as a result. Therefore, improving the lipids absorption efficiency by the intestines in piglets may improve intestinal energy supply of piglets and ameliorate weaning stress^8^. Dietary lipids are emulsified and disintegrated to produce free fatty acids, which enter cells via fatty acid transporters, including fatty acid translocase/CD36 and solute carrier family 27a^9^, and are transported into the endoplasmic reticulum via the fatty acid binding protein 3 (FABP3). Fatty acids transported into cells are re-synthesized into triglycerides and enter the bloodstream through the hepatic portal vein^10^. The previous study revealed that weaning piglets decrease the expression of fatty acid transport genes in intestinal epithelial cells^11,12^. Therefore, improving the efficiency of triglyceride synthesis in the intestines and increasing the energy supply may enhance the growth performance of piglets.

Valine is the fifth limiting amino acid in piglets^13^. Optimum dietary valine supplementation increases piglet weight gain, owing to its regulation of lipid metabolism^14^. However, the effects and mechanism of valine on intestinal lipid metabolism in piglets remain unclear and require investigations. Among the valine metabolites, 3-hydroxyisobutyrate (3-HIB) is the only substance that can survive mitochondrial oxidation and regulate cellular lipid metabolism, which plays an important role in regulating triglyceride synthesis^15,16^. Previous studies on human adipocytes have demonstrated that 3-HIB promotes the uptake of fatty acids, increasing the synthesis of triglycerides in cells^17^. Inhibition of 3-Hydroxyisobutyryl-CoA deacylase (HIBCH), a 3-HIB synthase, markedly reduces cellular triglyceride concentration^18^. Intake of 3-HIB in mice through drinking water or skeletal muscle injection accumulates diacylglycerol in the skeletal muscle^18^, indicating that 3-HIB plays a significant role in promoting triglyceride synthesis in the skeletal muscle cells of mice. Knockout of the HIBCH gene in mouse skeletal muscle cells considerably inhibited cell triglyceride synthesis^19^. However, knockout of the 3-HIB metabolic enzyme 3-Hydroxyisobutyryl-CoA dehydrogenase (HIBADH) to reduce the 3-HIB metabolic rate significantly increased triglyceride synthesis^15^, suggesting that 3-HIB may be used as a fatty acid transporter agonist, prompting the entry of more fatty acids into the cell for triglyceride synthesis. However, whether 3-HIB can affect intestinal health by regulating triglyceride synthesis in the intestinal cells of piglets has not been reported and remains to be studied.

Therefore, the present study explored the roles and molecular basis of valine and its metabolite (3-HIB) in lipid metabolism in the intestine of piglets. Our results may provide insights into the mechanisms underlying the beneficial effects of valine supplementation on triglyceride synthesis in the intestinal cells of piglets.

## Results

### Valine supplementation promoted triglyceride synthesis in IPEC-J2 cells

**Figure 1:**
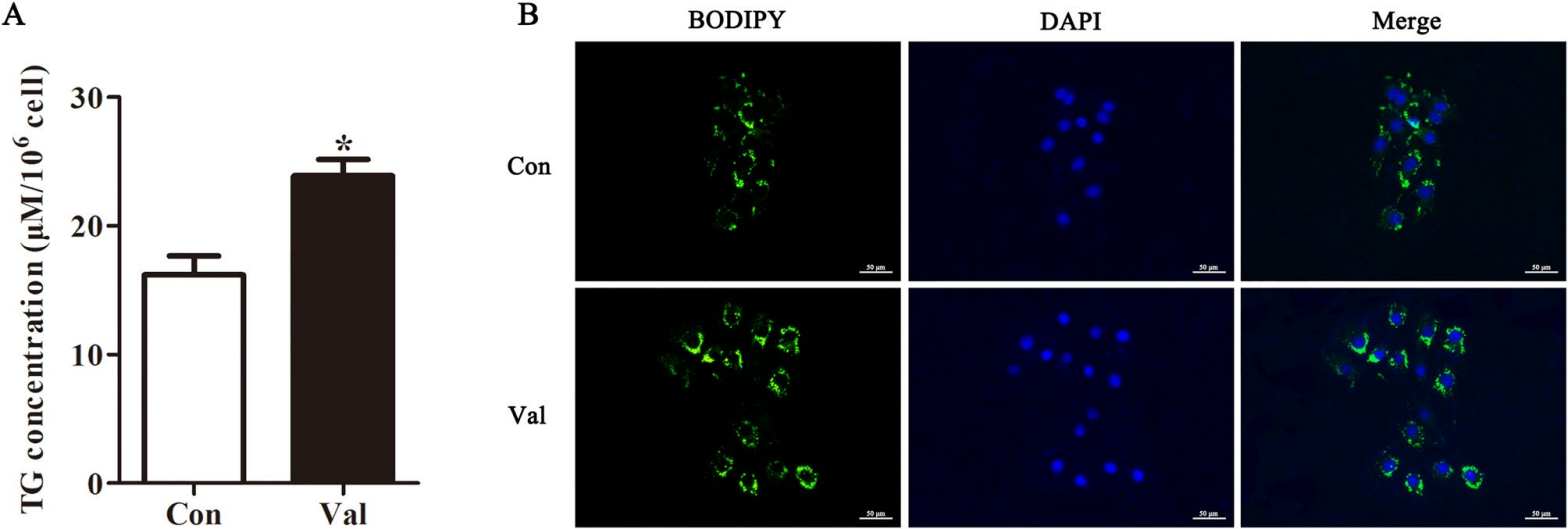
Valine supplementation promoted triglyceride synthesis in IPEC-J2 cells. Cells were incubated in 0.1 mM valine in medium (Con), and 0.9 mM valine in medium (Val), and culture medium was collected, and fat drop staining was performed after 48 h. (**A**) Triglyceride concentration in cells; (**B**) Immunofluorescent staining of lipid droplets. Scale bar represents 50 μm. TG: Triglyceride. All data with error bars represent the mean ± standard error of mean. ∗ *P* < 0.05.

The effect of valine supplementation on the concentration of triglyceride was presented in Fig. 1. Exposure of IPEC-J2 cells to 0.9 mM valine increased triglyceride concentration in IPEC-J2 cells compared with that in the Con group (Fig. 1A; *P* < 0.05). We further validated the synthesis and distribution of lipid droplets in cells stained with BODIPY 493/503. The result revealed that the lipid droplet content in cells increased with valine supplementation compared with that in the Con group (Fig. 1B), indicating that valine supplementation may increase triglyceride synthesis in IPEC-J2 cells.

### Valine supplementation increased the composition of fatty acids in IPEC-J2 cells

We compared the fatty acid composition in IPEC-J2 cells between the Con and Val groups (Table 1). A higher saturated fatty acid content in IPEC-J2 cells was observed in the Val group than that in the Con group (*P* < 0.05), including C17:0, C18:0, C20:0, and C21:0. Valine supplementation markedly increased the concentration of C18:1 in cells compared with that in the Con group (*P* < 0.05). Additionally, valine supplementation significantly increased the concentration of monounsaturated fatty acids C20:1 and C22:1 (*P* < 0.05). However, the concentration of polyunsaturated fatty acids (C18:3 and C20:3) in the medium were lower than those in the Con group (*P* < 0.05). No significant differences in the concentration of the other fatty acids were observed between the two groups (*P* > 0.05).

**Table 1:** Effect of valine supplementation on the composition of fatty acids in IPEC-J2 cells.

Fatty acids	Con	Val	SEM	*P* value
C14:0	0.71 ± 0.02	0.88 ± 0.11	0.06	0.215
C15:0	0.27 ± 0.01	0.27 ± 0.01	0.01	0.886
C16:0	12.59 ± 0.25	13.75 ± 0.37	0.33	0.061
C17:0	0.48 ± 0.01	0.52 ± 0.01	0.01	0.013
C18:0	10.02 ± 0.22	11.20 ± 0.18	0.29	0.015
C20:0	0.15 ± 0.01	0.18 ± 0.01	0.01	0.006
C21:0	0.13 ± 0.01	0.11 ± 0.01	0.01	0.004
C23:0	0.17 ± 0.01	0.13 ± 0.01	0.01	0.010
C16:1	1.74 ± 0.04	1.92 ± 0.17	0.09	0.353
C17:1	0.76 ± 0.02	0.78 ± 0.02	0.01	0.459
C18:1	43.39 ± 0.93	48.26 ± 1.11	1.26	0.028
C20:1	0.64 ± 0.02	0.78 ± 0.01	0.03	0.005
C22:1	0.17 ± 0.01	0.21 ± 0.01	0.01	0.009
C24:1	0.21 ± 0.01	0.20 ± 0.01	0.01	0.207
C18:2 n-6	2.65 ± 0.06	2.76 ± 0.04	0.04	0.209
C20:2	0.30 ± 0.01	0.34 ± 0.01	0.01	0.055
C18:3 n-6	0.18 ± 0.01	0.15 ± 0.01	0.01	0.042
C18:3 n-3	0.21 ± 0.01	0.18 ± 0.01	0.01	0.052
C20:3 n-6	0.82 ± 0.01	0.84 ± 0.03	0.01	0.700
C20:3 n-3	0.23 ± 0.01	0.20 ± 0.01	0.01	0.044
C20:4 n-6	4.64 ± 0.06	4.49 ± 0.09	0.06	0.233
C20:5 n-3	0.34 ± 0.01	0.32 ± 0.02	0.01	0.404
C22:6	1.87 ± 0.03	1.80 ± 0.04	0.02	0.201

Cells were incubated in 0.1 mM valine in medium (Con), and 0.9 mM valine in medium (Val). Results were presented as mean values with their standard errors.

### Valine supplementation increased 3-HIB concentration in the culture medium of IPEC-J2 cells

**Figure 2:**
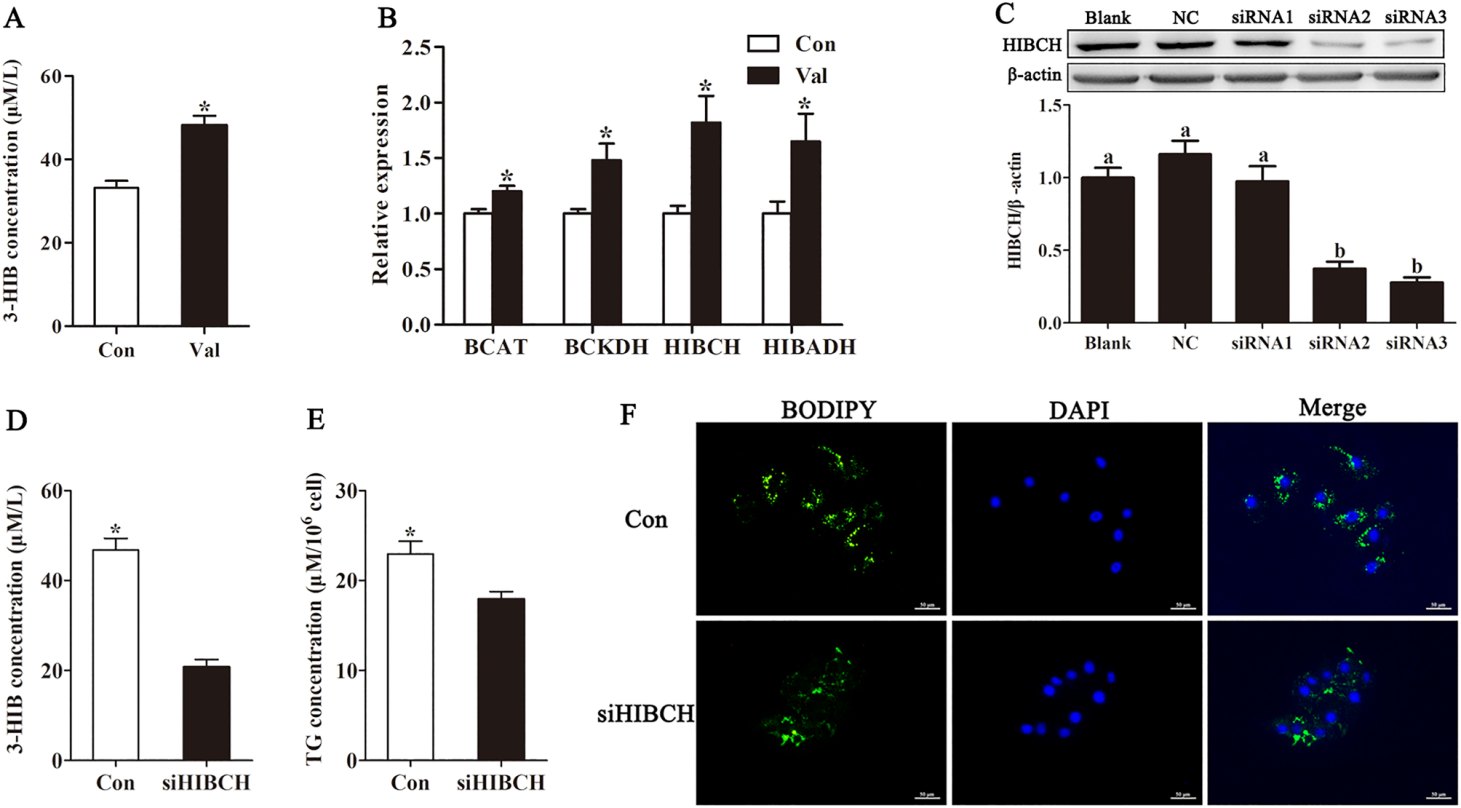
Valine promoted triglyceride synthesis in IPEC-J2 cells via 3-HIB. Cells were incubated in 0.1 mM valine in medium (Con) and 0.9 mM valine in medium (Val). The cells were transfected with 3-HIB synthase 3-hydroxyisobutyryl-CoA hydrolase HIBCH-siRNAs (siHIBCH) LipofectamineTM 3000. Culture medium collection, total RNA extraction, and fat drop staining was performed after 48 h. (**A)** 3-HIB concentration in medium with valine treatment; (**B)** 3-HIB metabolism-related gene mRNA expression; (**C**) Gene knockdown fficiency of candidate siRNAs in IPEC-J2 cells; (**D**,**E**) 3-HIB and TG concentration in the medium and cells after HIBCH silencing; (**F**) Immunofluorescent staining of lipid droplets. Scale bar represents 50 μm. 3-HIB: 3-hydroxyisobutyrate; HIBCH: hydroxyisobutyryl-CoA deacylase; TG: Triglyceride. All data with error bars represent the mean ± standard error of mean. ∗ *P* < 0.05.

3-HIB is an intermediate product in valine metabolism derived from the 3-hydroxyisobutyryl-coenzyme A hydrolase (HIBCH) and is catabolized by the 3-HIB dehydrogenase (HIBADH). Results revealed that increasing valine supplementation significantly increased the 3-HIB concentration in the culture medium (Fig. 2A; *P* < 0.05). Additionally, valine supplementation in the medium increased the mRNA expression of multiple enzymes involved in branched-chain amino acid catabolism, including branched-chain aminotransferase, branched-chain α-keto acid dehydrogenase, HIBCH, and HIBADH (Fig. 2B), indicating that 3-HIB may play an important role in the regulation of triglyceride synthesis.

### Valine promoted triglyceride synthesis in IPEC-J2 cells via 3-HIB

To determine the role of 3-HIB, RNA interference of HIBCH was used to investigate the function of 3-HIB in triglyceride synthesis in IPEC-J2 cells. The HIBCH protein expression is presented in Fig. 2C. siHIBCH-3 was the most effective silencer, thus was used in subsequent experiments. The 3-HIB concentration was significantly decreased (*P* < 0.05) when HIBCH was knocked down using siRNA (Fig. 2D). siHIBCH silencing using siRNA significantly decreased triglyceride concentration in the medium (*P* < 0.05) (Fig. 2E). siHIBCH knockdown in IPEC-J2 cells almost stopped the synthesis of lipid droplets (Fig. 2F), indicating that the number of fat droplets in cells decreased significantly.

### 3-HIB supplementation promoted triglyceride synthesis and related protein expression in IPEC-J2 cells

**Figure 3:**
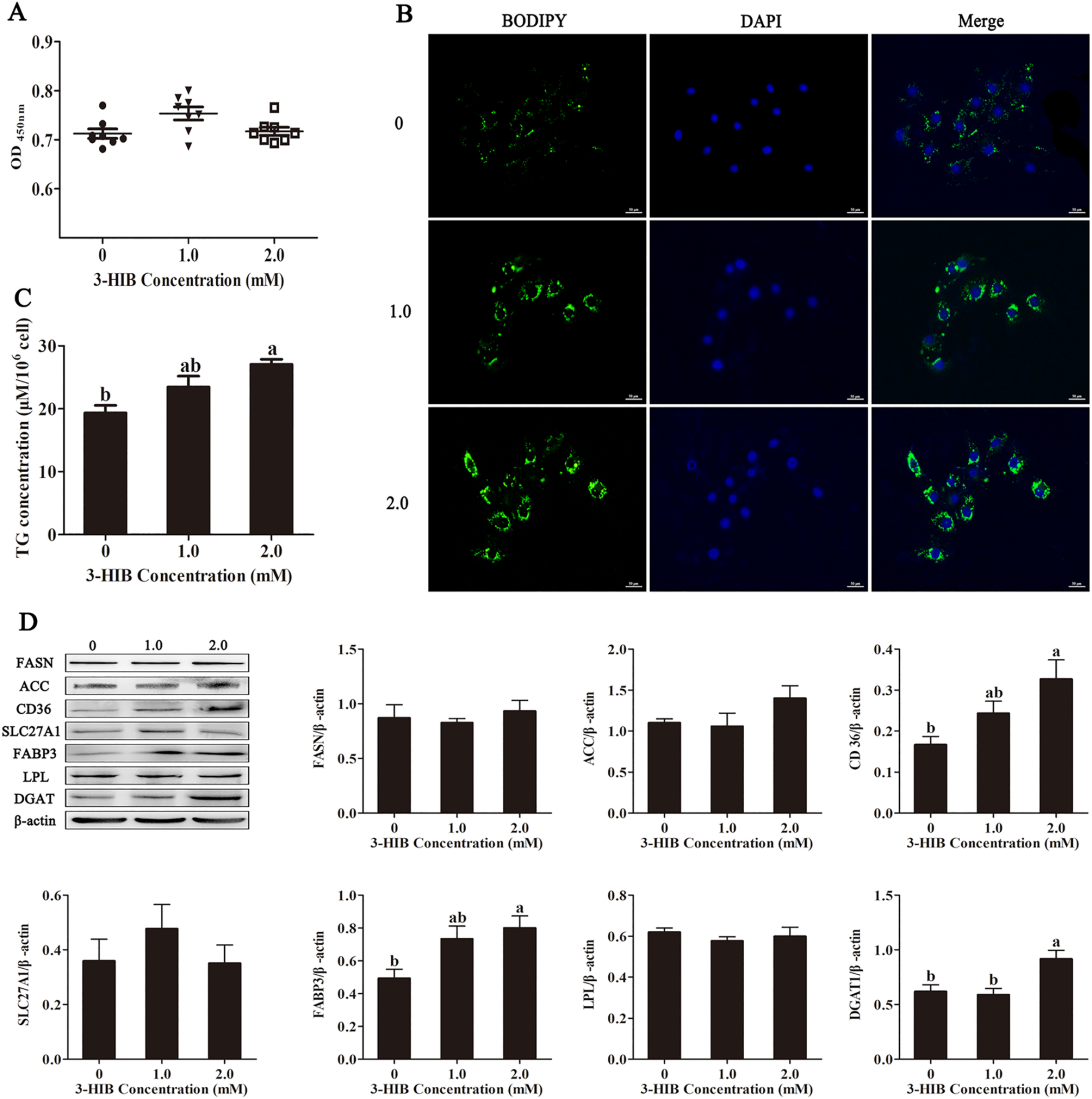
3-HIB supplementation promoted triglyceride synthesis and related protein expression in IPEC-J2 cells. Cells were incubated in different 3-HIB concentration groups (including 0, 1.0, and 2 mM). Culture medium and protein collection and fat droplet staining were performed after 48 h. (**A**) Cell proliferation was determined using a cell counting kit; (**B**) Lipid droplets in cells, scale bar represents 50 μm; (**C**) Triglyceride content in cells; (**D**) Western blot analysis of triglyceride synthesis related enzymes. TG: Triglyceride; CD36: fatty acid translocase / CD36; SLC27A: solute carrier family 27a; FABP3: fatty acid binding protein 3; FASN: fatty acid synthase; ACC: acetyl-CoA carboxylase; LPL: lipoprotein lipase; DGAT1: diacylglycerol transferase. All data with error bars represent the mean ± standard error of mean. ∗ *P* < 0.05.

Supplementing with 3-HIB significantly increased cell proliferation in IPEC-J2 cells compared with those in control groups (Fig. 3;* P* < 0.05). Treating with 3-HIB increased the intracellular triglyceride content (Fig. 3C; *P* < 0.05), consistent with results from cellular staining using the lipophilic dye BODIPY 493/503 (Fig. 3B). The protein expression levels of fatty acid transport-related enzymes, including CD36, FABP3 and DGAT1, were significantly upregulated (Fig. 3D; *P* < 0.05) when the cells were exposed to 2.0 mM 3-HIB.

### 3-HIB supplementation increased the composition of fatty acids in IPEC-J2 cells

A higher content of saturated fatty acids in IPEC-J2 cells was observed in the 3-HIB group than that in the control group (*P* < 0.05), including C14:0, C15:0, C17:0, and C21:0, consistent with the results of the valine-treated cells (Table 2). 3-HIB supplementation markedly increased the concentration of monounsaturated fatty acids in cells (*P* < 0.05) compared with that in the control group, including C16:1, C17:1, and C18:1. Polyunsaturated fatty acids, including C18:2, C18:3, C20:3, C20:4, and C20:5 were significantly higher in the 3-HIB supplementation group (*P* < 0.05) than those in the control group (Table 2).

**Table 2:** Effect of 3-HIB supplementation on the composition of fatty acids in IPEC-J2 cells.

Fatty acids	3-HIB concentration (mM)			SEM	*P* value
	0	1.0	2.0		
C14:0	0.87 ± 0.01^b^	0.92 ± 0.01^a^	0.90 ± 0.02^ab^	0.01	0.049
C15:0	0.27 ± 0.01^b^	0.31 ± 0.01^a^	0.32 ± 0.02^a^	0.01	0.001
C16:0	15.64 ± 0.25	16.30 ± 0.07	15.46 ± 0.42	0.19	0.168
C17:0	0.54 ± 0.01^b^	0.60 ± 0.01^a^	0.58 ± 0.01^a^	0.01	0.008
C18:0	12.89 ± 0.32	13.53 ± 0.01	12.46 ± 0.39	0.22	0.105
C20:0	0.20 ± 0.01	0.21 ± 0.01	0.18 ± 0.01	0.01	0.156
C21:0	0.12 ± 0.01^b^	0.14 ± 0.01^a^	0.13 ± 0.02^a^	0.01	0.009
C23:0	0.15 ± 0.01	0.16 ± 0.01	0.15 ± 0.02	0.01	0.109
C16:1	1.84 ± 0.03^b^	2.09 ± 0.01^a^	2.12 ± 0.03^a^	0.04	0.001
C17:1	0.81 ± 0.01^b^	0.94 ± 0.01^a^	0.93 ± 0.01^a^	0.02	0.001
C18:1	46.58 ± 0.07^b^	48.14 ± 0.42^b^	51.80 ± 1.23^a^	0.86	0.007
C20:1	0.80 ± 0.02	0.79 ± 0.01	0.82 ± 0.04	0.01	0.750
C22:1	0.20 ± 0.01	0.20 ± 0.01	0.21 ± 0.01	0.01	0.579
C24:1	0.25 ± 0.01^a^	0.25 ± 0.01^a^	0.22 ± 0.01^b^	0.01	0.021
C18:2 n-6	2.80 ± 0.04^b^	3.22 ± 0.02^a^	3.25 ± 0.05^a^	0.08	0.001
C20:2	0.43 ± 0.02	0.41 ± 0.01	0.39 ± 0.02	0.01	0.271
C18:3 n-6	0.14 ± 0.01^b^	0.16 ± 0.01^a^	0.17 ± 0.01^a^	0.01	0.002
C18:3 n-3	0.18 ± 0.01^b^	0.21 ± 0.01^a^	0.21 ± 0.01^a^	0.01	0.005
C20:3 n-6	0.85 ± 0.01^b^	0.97 ± 0.01^a^	0.98 ± 0.02^a^	0.02	0.001
C20:3 n-3	0.23 ± 0.01^b^	0.26 ± 0.01^a^	0.25 ± 0.01^a^	0.01	0.005
C20:4 n-6	5.54 ± 0.04^b^	6.04 ± 0.01^a^	5.87 ± 0.14^a^	0.09	0.017
C20:5 n-3	0.35 ± 0.01^c^	0.40 ± 0.01^b^	0.43 ± 0.01^a^	0.01	0.001
C22:6	2.29 ± 0.03	2.43 ± 0.01	2.33 ± 0.07	0.03	0.130

Results were presented as mean values with their standard errors. Means not sharing the same letter are different (*P* < 0.05).

## Discussion

Branched chain amino acids (BCAAs; leucine, isoleucine and valine) play important role in protein synthesis, cell proliferation, cell metabolism, and signal pathway activation^20,21^. Valine is the third-most limiting amino acid in lactating sows^22^ and the fifth limiting in piglets^13^. Piglets with insufficient supply of valine appear to be a reduction in feed intake^23^. On the other hand, previous studies have demonstrated that an increased supply of leucine will stimulate the activity of BCAAs metabolic enzymes and may therefore increase the catabolism of valine^23^ and lead to the deficiency of valine in piglets. It is necessary to further analyze the regulatory effect and mechanism of valine on physiological function of piglets. Previous studies from our laboratory indicated that optimal valine supplementation in the diet of sows could improve the weaning weight of piglets by increasing the milk fat synthesis in sows^24^, indicating that valine is closely related to fat metabolism in sow mammary glands. Recent studies in humans and rats have highlighted that the blood valine level may indicate whether lipid metabolism is balanced^25,26^. Although valine has been extensively studied considering the intestinal health of piglets^27^, the mechanism by which valine regulates the intestinal health of piglets is unclear. The effect of valine on intestinal cell lipid metabolism in piglets is unknown.

Our present study revealed that valine significantly increased the levels of triglycerides and lipid droplets in IPEC-J2 cells, consistent with the increase in triglycerides and lipid droplets in porcine mammary epithelial cells in our previous study^28^. Triglycerides are formed through the dehydration condensation reaction of glycerol and long-chain or medium-chain fatty acids; therefore, the type and amount of fatty acids play a role in the amount of triglycerides produced. Analysis of the fatty acid composition in cells revealed that the concentration of saturated fatty acids and unsaturated fatty acids in cells increased considerably in the valine group. Previous studies have demonstrated that long-chain fatty acids in cells depend on extracellular uptake through targeted metabolomics analysis in the plasma and milk^29^. Dietary valine supplementation considerably reduced the content of some fatty acids in the plasma, whereas increasing that in the colostrum^22^, suggesting that valine may affect the milk fat rate by regulating the transport of fatty acids in the blood by mammary gland cells. Therefore, based on the present study results, valine may promote the fatty acid uptake efficiency of IPEC-J2 cells. However, the underlying regulatory mechanism remains unclear.

Valine is first converted to α-ketoisovaleric acid by branched-chain amino acid metabolic enzymes, and the branched-chain α-keto acid dehydrogenase enzyme complex catalyzes the oxidative decarboxylation of α-ketoisovaleric acid^30^. The branched-chain α-ketoacid dehydrogenase complex exists in the mitochondrial inner membrane; therefore, valine metabolites are converted to ATP in the mitochondria through the tricarboxylic acid cycle^31^. However, Jang et al. (2016) reported that 3-HIB, a unique valine metabolite, can be released outside the cells through the mitochondrial membrane^15^, providing a research gap on valine lipid metabolism regulation. In the present study, we found that the 3-HIB concentration in the valine supplementation group was markedly increased, whereas the mRNA expression of the 3-HIB synthase HIBCH was markedly upregulated. siRNA interference was used to investigate the 3-HIB function in valine-induced triglyceride synthesis. Knocking down of HIBCH using siRNA markedly decreased the triglyceride concentration of the medium. The concentration of lipid droplets was confirmed to be higher in IPEC-J2 cells by lipophilic dye BODIPY 493/503 compared with that in related controls, suggesting that 3-HIB plays an important role in regulating cellular triglyceride synthesis, consistent with the results of previous studies. Zoltan and Michael (2018) examined the 3-HIB function and found that the 3-HIB content in skeletal muscle increased by 1.6-fold, and the triglycerides and diacylglycerol contents in skeletal muscle significantly increased after mice were fed 3-HIB-containing drinking water for 2 weeks^18^. Additionally, the 3-HIB direct injection into mice caused diacylglycerol accumulation in the skeletal muscle. There are no reports on the 3-HIB regulation in triglyceride synthesis in IPEC-J2 cells, and few studies have investigated 3-HIB analogs. For example, 3-hydroxybutyrate (3-HB) participates in de novo synthesis of fatty acids as a precursor of milk fat synthesis in ruminants^32^. Song et al. (2020) revealed that 3-HB supplementation significantly increased the expression of fatty acid synthesis-related genes in bovine mammary epithelial cells by analyzing the cell transcriptome^33^. β-hydroxy-β-methylbutyric acid (HMB) is a key intermediate product in leucine metabolism^34^. Previous studies have confirmed that HMB addition to the diet of sows from 35 days of gestation to delivery significantly increases milk fat content during lactation^35^. The present study revealed that 3-HIB could significantly increase the triglyceride content in IPEC-J2 cells by adding 3-HIB to the cell culture medium, consistent with the results of fluorescent staining of fat droplets.

The concentration of triglycerides in pig intestinal epithelial cells is affected by the concentration of fatty acids, and de novo synthesis and transport of fatty acids in cells play an essential role^36^. Acetyl coenzyme A in cells is catalyzed by acetyl CoA carboxylase to synthesize malonyl coenzyme A, and fatty acids are subsequently synthesized and catalyzed by the fatty acid synthase enzyme^37^. Furthermore, long-chain fatty acids are absorbed into cells through active transport mediated by CD36, SLC27A, and FABP3. Finally, de novo fatty acids are transported in cells to synthesize triglycerides catalyzed by diacylglycerol acyltransferase^9^. Western blotting revealed increased CD36, FABP3, and DGAT1 protein expression in IPEC-J2 cells, indicating that an increase in triglyceride synthesis may be related to the transport of fatty acids by cells, consistent with the fatty acid composition in the cells. The concentrations of most long-chain fatty acids in the 3-HIB group, including C17:0, C21:0, C16:1, C17:1, and C18:1 increased considerably. In similar studies in rat skeletal muscle cells, the knockout of the HIBCH gene in cells considerably inhibited the transport of fatty acids. Contrastingly, knockout of the 3-HIB metabolic enzyme HIBADH decreased the 3-HIB metabolic rate and markedly increased the intake of fatty acids^15^, suggesting that 3-HIB may promote triglyceride synthesis through the transportation of cellular fatty acids. Taken together, these data demonstrate that valine in IPEC-J2 cells can be catabolized into 3-HIB, which may act as a paracrine factor to stimulate fatty acid uptake of IPECJ2 cells^15^.

## Conclusion

Our results demonstrate that valine supplementation in the culture medium stimulates triglyceride synthesis in IPEC-J2 cells by increasing the 3-HIB concentration, which may promote fatty acid transport via upregulation of the fatty acid transporter mechanism. These findings provide new insights into the mechanisms through which valine participates in lipid metabolism. The 3-HIB application may improve the intestinal health of piglets and could be developed as a feed additive.

## Materials and methods

### Reagents

Porcine intestinal epithelial (IPEC-J2) cells were purchased from Zhenzhou Aibokang Science and Technology Ltd. Valine-free Dulbecco's Modified Eagle Medium/Nutrient Mixture F-12 (DMEM/F12), fetal bovine serum (FBS), antibiotics, trypsin/EDTA, and sterile phosphate-buffered saline (PBS) were procured from Invitrogen (Calsbad, CA). Plastic culture plates and centrifuge tubes were manufactured by Corning Inc (Corning, NY). Valine, 3-HIB, epidermal growth factor (EGF) and BODIPY (493/503) were obtained from Sigma-Aldrich. The triglyceride and 3-HIB kits were purchased from Applygen echnologies Inc (Beijing, China) and Nanjing Jiancheng Bioengineering Institute (Nanjing, Jiangsu, China), respectively. Protein expression of anti-FASN (ab128870), anti-SLC27A1 (ab81875), anti-FABP3 (ab133585), anti-LPL (ab91606), anti-DGAT1 (ab181180) were purchased from Abcam (Cambridge, UK). Anti-ACC (#3662 s), anti-CD36 (#74,002), anti-rabbit IgG (#7074) and anti-mouse IgG (#7076) antibodies were obtained from Cell Signaling Technology (Beverly, MA). Anti-HIBCH (sc-515355) and anti-β-actin (anm40032) antibodies were purchased from Santa Cruz Biotechnology (Santa Cruz, CA) and Amyjet Scientifific (Wuhan, China).

### Cell culture and treatment

Based on a method described in a previous study^38^, IPEC-J2 cells in the logarithmic growth phase were selected and cultured in cell culture dishes containing growth medium (including 90% DMEM/F12, 10% FBS, 5 ng/mL EGF and 1 × PSN antifungal/antibiotics) at 37 °C under 5% CO_2_, and the medium was changed every 2 days. The IPEC-J2 cells were passaged using 0.25% trypsin-ethylenediaminetetraacetic acid (EDTA) after reaching 80–90% confluency. Next, cells were reseeded at 6.0 × 10^3^ cells/well in 96-well plates or 2.5 × 10^5^ cells/well in 6-well plates. Valine or 3-HIB was added to the culture medium to determine their effects on triglyceride synthesis and fatty acid composition.

### Triglyceride and 3-HIB concentration

IPEC-J2 cells were seeded into 6-well cell culture plates, as described above^38^, with 2 mL/well culture medium. To study the effects of valine or 3-HIB supplementation on triglyceride synthesis of IPEC-J2 cells, cell culture medium was prepared with valine-free DMEM/F12, valine (final concentrations of 0.1 and 0.9 mM) or 3-HIB (final concentrations 0, 1.0 and 2.0 mM, valine concentration of 0.1 mM) was added into the medium and the cells were cultured for 48 h before measuring valine and 3-HIB concentrations. The final 3-HIB concentration referred to previous studies in Nature Medicine^15^. The effect of valine or 3-HIB supplementation on the triglyceride concentration in cells and 3-HIB concentration in the medium was measured using commercial triglyceride assay kits.

### BODIPY staining of lipid droplets

IPEC-J2 cells were cultured in six-well plates with different valine (0.1 and 0.9 mM) or 3-HIB (0, 1.0, and 2.0 mM) concentrations. After the cells were cultured for 48 h, the medium was removed, and the cells were washed thrice with phosphate-buffered saline (PBS). The BODIPY 493/503 was used to monitor the content of neutral lipids in IPEC-J2 cells according to a previously described method^39^. Briefly, the cells were fixed in 4% paraformaldehyde for 30 min, and were stained with BODIPY 493/503 lipophilic fluorescence dye (final concentration 1 μg/mL) for 15 min in a dark area at room temperature (about 25 °C). Cells were overlaid with 4, 6-diamidino-2-phenylindole reagent Hoechst 33,258 (Beyotime, Shanghai, China), incubated in the dark for 10 min, washed with PBS buffer, and mounted with antifade mounting medium (Beyotime, Shanghai, China). Finally, imaging was done using a fluorescence microscope (NIS-Elements, Nikon, Japan).

### Analysis of the composition of fatty acids

IPEC-J2 cells were cultured in six-well plates with different valine (0.1 and 0.9 mM) or 3-HIB (0, 1.0, and 2.0 mM) concentrations for 48 h. Cell samples were collected after digestion with 0.25% trypsin–EDTA. The composition of fatty acids in the cells was analyzed using gas chromatography, as described in a previous study^40^. Briefly, cells were extracted and purified in chloroform and methanol at a 2:1 ratio. Next, the extract was saponified with sodium methylate, and esterified with 1% H_2_SO_4_ in anhydrous methanol for 2 h at 70 °C. The obtained fatty acid methyl ester was extracted using hexane, and 1 μL of sample was injected into the column using an automatic injector at a 5:1 split ratio. The fatty acid methyl esters were separated on a 30 m × 320 μm × 0.25 μm DB-23 capillary column and helium was used as the carrier gas. The injector and detector temperature were maintained at 250 and 230 °C, respectively. The initial oven temperature was 50 °C, 175 °C for 1 min, and increased to 230 °C at a rate of 4 °C/min. The results are presented as concentrations of the target fatty acids.

### Transient transfection and HIBCH siRNA

Three candidate RNA (siRNA) targeting the HIBCH mRNA coding region of and a negative control siRNA were designed and purchased from GenePharma, Shanghai, China, to determine the 3-HIB function. IPEC-J2 cells were seeded at 1 × 10^5^ cells/well in six-well plates containing induction medium and cultured for 24 h. Transfection was conducted using Lipofectamine 3000 reagent RNAiMAX (Invitrogen, Carlsbad, CA, USA) according to the manufacturer's protocols. We measured the specificity and effectiveness of IPEC-J2 cell siRNAs by determining the HIBCH protein expression after siRNA transfection for 48 h. siHIBCH-3 was the most expressed protein; thus, was selected for further analysis.

### Western blot analysis

After 48 h of culture, the cells were collected for protein analysis using the western blotting method based on a previous study^41^. First, cells were lysed using RIPA buffer (Beyotime, Beijing, China), total protein was measured by centrifugation, and the protein concentration in the supernatant fluid was determined using a BCA protein assay kit (Beyotime, Beijing, China) following the manufacturer's instructions. Next, 20 μg of protein samples were separated by sodium Dodecyl Sulphate–Polyacrylamide Gel Electrophoresis and transferred onto a polyvinylidene difluoride (PVD) membrane (Beyotime, Beijing, China). According to the size of target proteins, the blots were cut prior to hybridisation with antibodies during blotting. PVD membranes were incubated with primary antibodies overnight at 4 °C after being locked using a Western Quick Block kit (Beyotime, Beijing, China). Next, the PVD membranes were incubated with secondary antibodies for 1 h at room temperature (about 25 °C). After washing thrice with Tris-Buffered Saline Tween-20 the membranes were visualized using a chemiluminescent horseradish peroxidase substrate (Millipore, Billerica, MA) and VersaDoc imaging system (Bio-Rad, Hercules, CA). Band densities were calculated using Quantity One software (Bio-Rad Laboratories) and normalized to the β-actin density.

### Statistical analyses

All data are presented as mean ± standard error of mean. Data were analyzed using the Statistical Package for Social Sciences software (v. 22.0, SPSS; IBM Company, Chicago, IL). Student's t-test was used to detect significant differences between the valine treatment groups. For the 3-HIB test, significant differences in assay values were evaluated using a one-way analysis of variance, and Tukey's test was used to determine the differences among the groups. Factors with *P* values of < 0.05 were considered significantly different.

## Supplementary Information

Supplementary Information.

## Data Availability

The data used to support the findings of this study are available from the corresponding author upon request.

## Abbreviations

IPEC-J2 cells: Porcine intestinal epithelial cells
3-HIB: 3-Hydroxyisobutyrate
HIBCH: Hydroxyisobutyryl-CoA deacylase
HIBADH: 3-Hydroxyisobutyryl-CoA dehydrogenase
CD36: Fatty acid translocase/CD36
SLC27A: Solute carrier family 27a
FABP3: Fatty acid binding protein 3
FASN: Fatty acid synthase
ACC: Acetyl-CoA carboxylase
LPL: Lipoprotein lipase
DGAT1: Diacylglycerol transferase
