# Supplementary material for: Molecular mechanism of valine and its metabolite in improving triglyceride synthesis of porcine intestinal epithelial cells
Source: Sci Rep. 2023 Feb 20;13:2933. doi: 10.1038/s41598-023-30036-w (PMC9941501; doi:10.1038/s41598-023-30036-w)
Supplement: Supplementary file 1 — Supplementary Information. [file 41598_2023_30036_MOESM1_ESM.pdf]

Molecular mechanism of valine and its metabolite in improving triglyceride synthesis of porcine intestinal epithelial cells

Mengmeng Xu [1, #], Long Che [1, #, *], Lizhu Niu [1], Liuzhen Wang [1], Mengyun Li [1], Dongfeng Jiang [1], Hongyu Deng [1], Wen Chen [2], Zongyong Jiang [3]

[1] College of Animal Science and Technology, Henan University of Animal Husbandry and Economy, Zhengzhou, Henan 450046, China

[2] College of Livestock Husbandry and Veterinary Engineering, Henan Agricultural University, No. 15 Longzi Lake University Campus, Zhengzhou, 450046, China.

[3] Institute of Animal Science, Guangdong Academy of Agricultural Sciences, Guangzhou, Guangdong 510640, China.

[#] These authors contributed equally to the work

[*] Corresponding author: Long Che

Email address: chelong1989@126.com

Mailing address: College of Animal Science and Technology, Henan University of Animal Husbandry and Economy, No.6 North Longzihu Road, Zhengdong New District, Zhengzhou, 450046, China

post code: 450046

Running title: 3-HIB promotes triglyceride synthesis of IPEC-J2 cells

Supplementary Material. Original images of the western blot. The final 3-HIB concentrations referred to previous studies in Nature Medicine (Jang 2016). The cell proliferation and expression of some proteins were significantly decreased in the high 3-HIB concentration group. Therefore, 0 mM, 1.0 mM and 2.0 mM were selected as the final concentration of 3-HIB in this article.

Figure 2

HIBCH

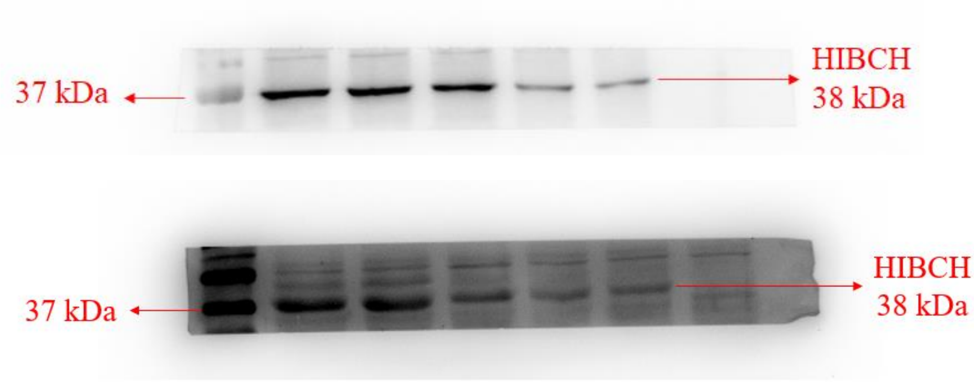

β-actin

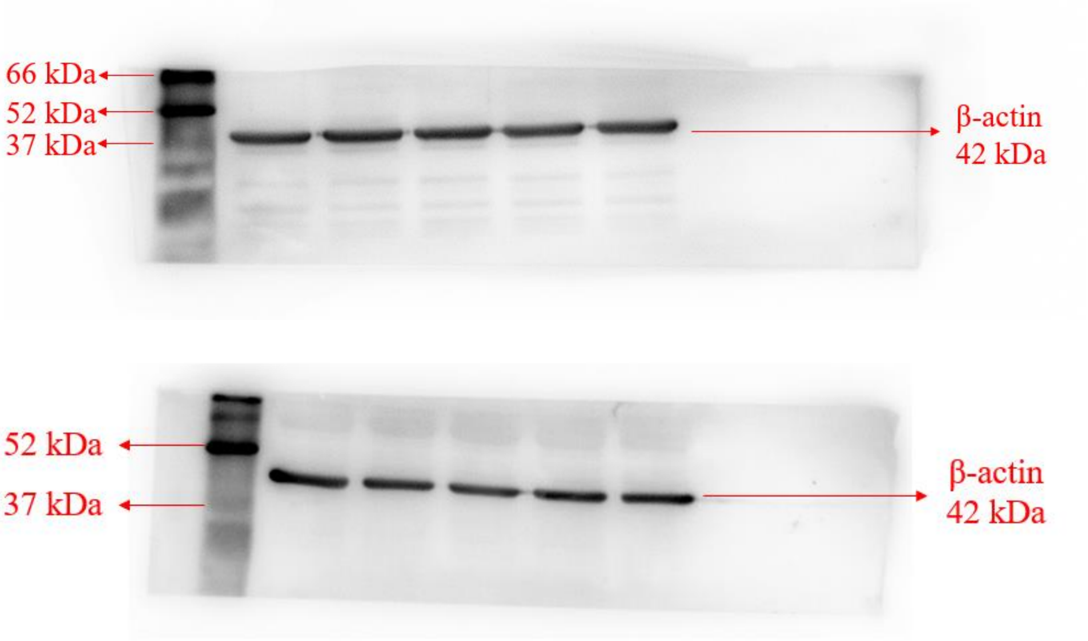

Figure 3

FASN

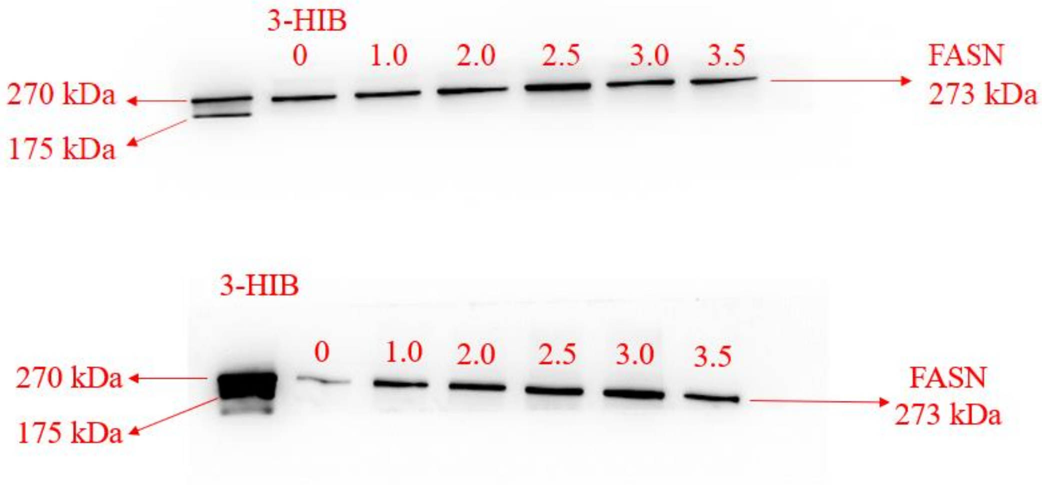

ACC

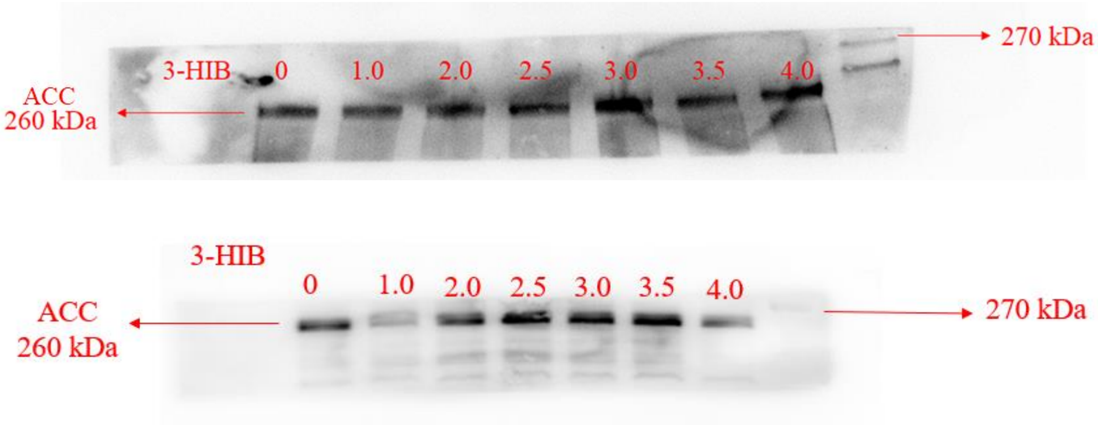

CD36

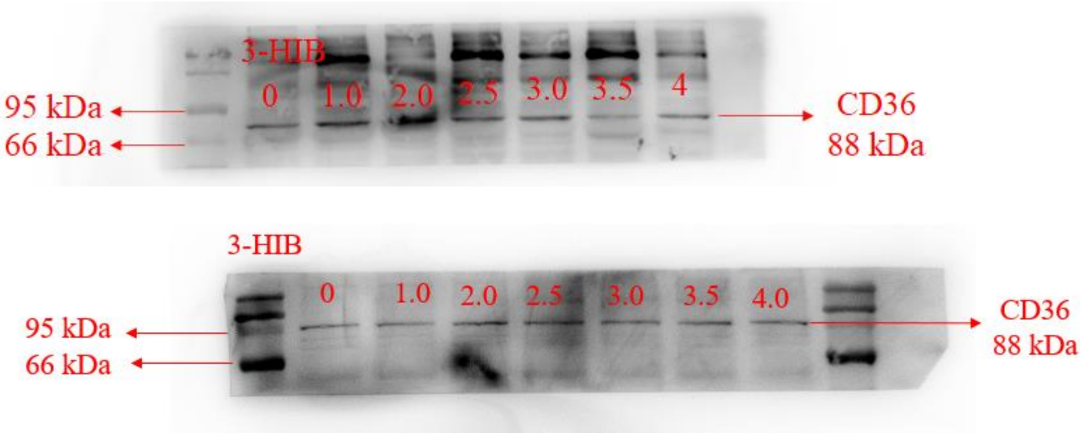

SLC27A1

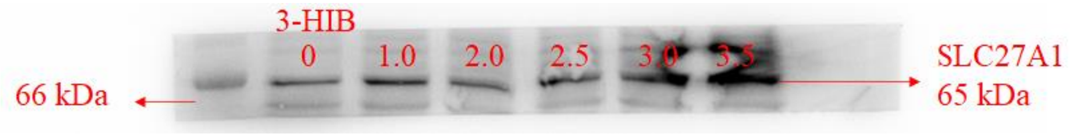

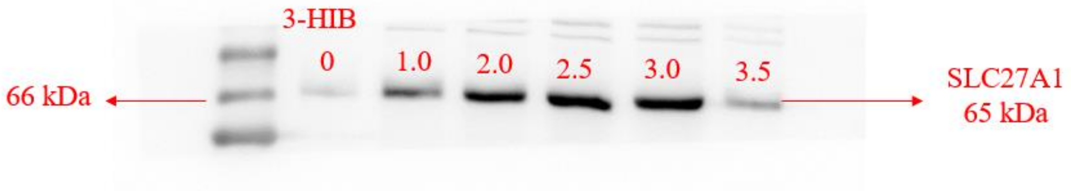

FABP3

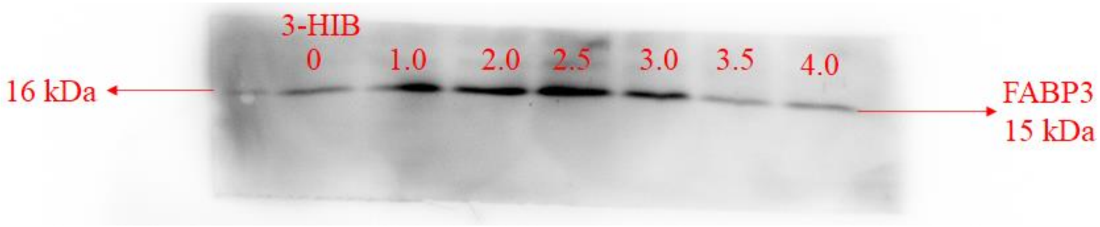

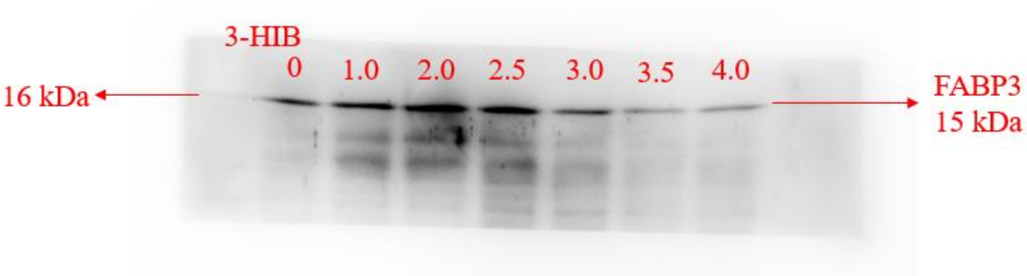

LPL

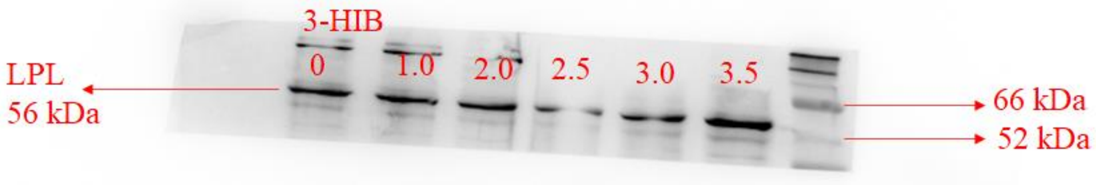

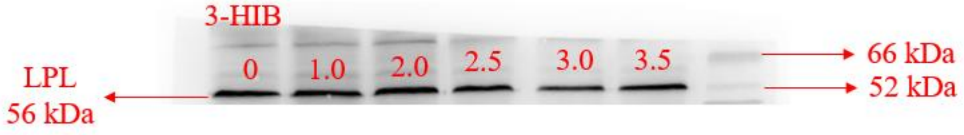

DGAT

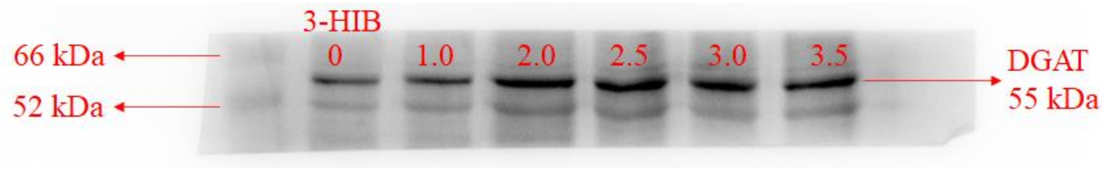
3-HIB
0
1.0
2.0
2.5
3.0
3.5
66 kDa
52 kDa
DGAT
55 kDa

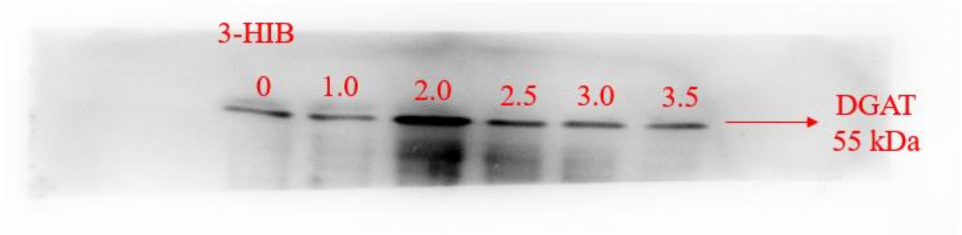
3-HIB
0
1.0
2.0
2.5
3.0
3.5
DGAT
55 kDa

β-actin

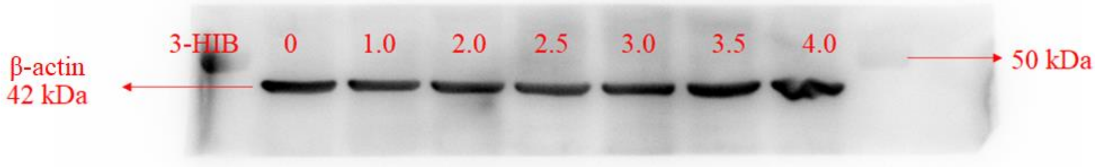
3-HIB
0
1.0
2.0
2.5
3.0
3.5
4.0
β-actin
42 kDa
50 kDa

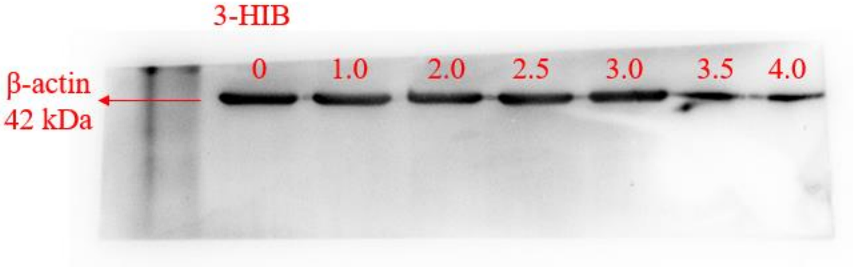
3-HIB
0
1.0
2.0
2.5
3.0
3.5
4.0
β-actin
42 kDa
